# A model of *Helicobacter* infection using an artificial liver constructed by a radial-flow bioreactor

**DOI:** 10.1007/s13577-026-01411-2

**Published:** 2026-07-01

**Authors:** Kyoko Ito, Haruka Maehashi, Tomohiro Kato, Tomokazu Matsuura

**Affiliations:** 1 Center for Preventive Medicine, The Jikei University Hospital, 3-19-18 Nishi-Shimbashi, Minato-Ku, Tokyo, 105-8471 Japan; 2 Department of Laboratory Medicine, The Jikei University School of Medicine, Minato-Ku, Tokyo, Japan; 3 Sasaki Foundation Shonan Health Examination Center, Hiratsuka-Shi, Kanagawa Japan

**Keywords:** Apoptosis, Bioartificial liver, *Helicobacter pylori*, Radial flow bioreactor

## Abstract

*Helicobacter* DNA has been detected in liver tissues from patients with hepatocellular carcinoma and other liver diseases, suggesting a potential involvement of *Helicobacter* infection in hepatic pathogenesis. However, whether *Helicobacter pylori* (*H. pylori*) can infect human hepatocytes and how such infection affects hepatocyte structure and function remain unclear. In this study, we used a three-dimensional (3D) bioartificial liver model reconstructed using radial-flow bioreactor technology to investigate the ability of *H. pylori* to infect hepatocytes and to characterize the cellular responses induced by infection. Histological analysis and electron microscopy demonstrated that *H. pylori* adhered to the hepatocyte surface and penetrated intercellular spaces. Infection significantly induced a threefold increase in apoptosis, accompanied by upregulation of TNF-α (1.5-fold) and activation of NF-κB (1.6-fold) (all *P* < 0.05). In addition, PCNA expression significantly decreased to approximately 60% of control levels (*P* < 0.05), whereas Akt activation was enhanced and β-catenin was predominantly localized in the cytoplasm. No significant differences were observed in Ki-67, IL-8, Ets-1, or c-Met expression compared with controls. These findings demonstrate that *H. pylori* can infect the 3D liver model and induce apoptosis and signaling alterations in hepatocytes, suggesting a potential pathological impact on the liver. Further studies are required to determine whether similar effects occur in the human liver in vivo.

## Introduction

Over the three decades since the discovery of *Helicobacter pylori* (*H. pylori*), at least 42 new *Helicobacter* species have been identified [1]. Several of these species have been detected in the livers of mice and dogs, where they induce inflammatory cell infiltration and histological changes in liver tissue [2–4]. In humans, *Helicobacter* DNA has been detected in the liver tissue of patients with various liver diseases, primarily through molecular methods, suggesting a potential role for *Helicobacter* species in the progression of liver pathology [5–8]. Epidemiological studies and meta-analyses have also indicated an association between *H. pylori* infection and an increased risk of hepatobiliary diseases disorders [9, 10]. Furthermore, patients co-infected with hepatitis C virus (HCV) and *H. pylori* have been reported to have a higher risk of progressing to advanced cirrhosis compared to those infected with HCV alone [11]. These findings suggest that *H. pylori* may exacerbate liver damage and fibrosis initiated by other factors, such as hepatitis viruses.

In our previous study [8], DNA sequencing and immunostaining results indicated that *H. pylori*, or a closely related *Helicobacter* species, can be present in human liver tissue. Our subsequent in vitro studies showed that *H. pylori* adhered to hepatocytes, invaded the cells, and survived intracellularly over an extended period. This infection was also found to modulate hepatocyte apoptosis and DNA synthesis [12, 13], suggesting that *H. pylori* can directly influence hepatocyte homeostasis [12, 13].

In the present study, we aimed to investigate the capacity of *H. pylori* to infect hepatocytes in a three-dimensional (3D) culture environment and to characterize the resulting impact on cellular inflammatory responses. A key feature of this study is the use of a radial flow bioreactor (RFB), which enables the construction of 3D cell cultures that more closely mimic in vivo tissue architecture compared to conventional 2D cultures [14]. This system reproduces cell polarity, allowing for a more detailed investigation of the infection routes and the physiological effects of *Helicobacter* on hepatocytes. By utilizing this model, we aimed to: (1) characterize the invasive behavior of *H. pylori* in 3D-cultured hepatocytes, (2) assess the impact of infection on the hepatocyte survival, and (3) identify cellular signaling pathways triggered by the infection.

## Materials and methods

### 3D hepatocyte culture in a radial flow bioreactor (RFB)

A 3D cell culture system was constructed using 5 mL RFB (RA-5; ABLE, Tokyo, Japan) filled with cellulose beads (Asahi Kasei, Tokyo, Japan), a mass flow controller (RAD925, ABLE), and a closed-circuit reservoir, as described previously [15]. The system was filled with ASF 104N medium (AJINOMOTO, Cyuo-ku, Tokyo, Japan) containing 2% fetal bovine serum, 0.1% of D-glucose and 0.0125% of C^13^-glucose. FLC-4 cells (RRID:CVCL_D204) [16] is a human hepatocellular carcinoma-derived cell line established and have been maintained at The Jikei University School of Medicine that maintains functions similar to normal hepatocytes. For the experiment, 1 × 10^7^ cells were infused into the reservoir to facilitate attachment to the cellulose beads, and a 3D cell culture was constructed for 2 weeks until the cell culture reached approximate confluence. The culture medium was changed manually to maintain optimal glucose, lactate, CO_2_ and pH levels [15]. These levels were monitored as follows: 0.5 ml sample of culture medium was collected, and glucose, lactate, and pH were measured once or twice daily, in the morning and/or evening, using GLUTEST Sensor (ARKRAY, Inc. Minneapolis, MN, USA and SKK/SANWA KAGAKU KENKYUSYO, Co.,Ltd. Nagoya-shi, Aichi, Japan) for glucose and lactate measurement, and LAQUAtwin Compact pH Meter (HORIBA Advanced Techno, CO., Ltd., Miyanohigashi-cho Kisshoin Minami-ku, Kyoko, Japan) for pH measurement. Simultaneously, a dedicated gas collection bag was used to collect air from inside the reactor and measured the CO_2_ level by POCone (Otsuka Pharmaceutical Co., Ltd., Chiyoda-ku, Tokyo, Japan). Glucose, lactate, pH, and CO_2_ were maintained approximately 200–300 mg/dL, 5–12 mmol/L, 7.3–7.5, and 5%, respectively.

### *H. pylori *infection protocol

*H. pylori* strain NCTC 11637 (RRID:TXID208960), a virulent strain positive for the virulence factors CagA and VacA, was kindly provided by Dr. Yoshio Yamaoka and Dr. David Y. Graham of Baylor College of Medicine (Houston, Texas, USA). Bacteria (1 × 10^12^ colony forming units [CFUs]) were cultured on blood agar plates consisting of brain–heart infusion agar (Difco, Sparks, MD, USA) and 7% horse blood for 2 days before infection in a CO_2_ incubator with 12% CO_2_ at 37 ℃. The bacteria were suspended in cell culture medium as described above and adjusted to an appropriate concentration before use.

The bacterial concentration was standardized by measuring the optical density at 600 nm. Overall, 15 mL of 1.1 × 10^9^ /mL bacterial suspension with culture medium was inserted into the reserve. CFUs were measured as described previously [12] and found to be 7.5 × 10^9^, confirming that the multiplicity of infection was approximately 100. Moreover, 15 mL culture medium without bacteria was used as the control. After 24 and 48 h of incubation, 3D cell culture was collected, stored at –80 °C, frozen in optimal cutting temperature compound for cryo-sections, and fixed in 2.5% glutaraldehyde for electron microscopy and 10% buffered formalin.

### Histological analysis

Three samples were randomly collected from both the inlet and outlet of the RFB reservoir for histological assessment. Morphology was assessed using hematoxylin and eosin (H and E) staining. Paraffin-embedded and formalin-fixed 3D cell culture Sects. (4 μm thick) were stained with H and E.

Immunohistochemistry (IHC) for *H. pylori* was performed to assess the infection status. After de-embedding and blocking with 3% MtOH in H_2_O_2_ for 10 min, antigen retrieval was performed using Liberate Antibody Binding solution (Polysciences Inc., Warrington, PA, USA) for 5 min at room temperature. After washing three times with phosphate-buffered saline (PBS) for 5 min, 1:50 diluted rabbit polyclonal anti-*H. pylori* antibody (DAKO, Kyoto, Japan) in 5% bovine serum albumin with PBS was applied to the slides and incubated for 60 min at room temperature. Secondary antibody and enhancement steps were performed according to the instructions of ENVISION kit/HRP(DAB) (DAKO, Kyoko, Japan).

### Electron microscopy

Both transmission electron microscopy (TEM) and scanning electron microscopy (SEM) were conducted according to standard protocols at the Molecular Cell Biology Facility of The Jikei University School of Medicine [17]. Four samples were obtained from each condition (with or without *H. pylori*), including samples collected from both the inlet (*n* = 2) and outlet (*n* = 2) of the reservoir.

### Immunohistochemical and immunofluorescence staining

To assess apoptosis, cell proliferation and the activation of intracellular signaling pathways, histological staining was performed. Apoptosis was detected using the TUNEL assay, while cell proliferation was evaluated by Ki67 immunohistochemistry. Additionally, the expression and localization of Akt, β-catenin, and c-Met were analyzed by immunofluorescence staining.

The TUNEL assay was performed in 4 µm of formalin-fixed and paraffin-embedded sections using the In Situ Cell Death Detection Kit, POD (Roche Applied Science, Tokyo, Japan). Four representative sections were obtained from both *H. pylori*-infected and uninfected control cells for TUNEL assay. For the evaluation of the TUNEL assay, cells with distinct brown DAB (3,3'-Diaminobenzidine) staining in their nuclei were defined as TUNEL-positive, while cells whose nuclei showed only hematoxylin counterstaining without any observable DAB signals were identified as negative. To ensure accuracy and distinguish specific nuclear signals from non-specific background staining, this determination was performed under high magnification (× 400). Data were analyzed and compared using LI, which is expressed as the percentage of nuclei with signals among 100 nuclei in a randomly chosen area.

IHC was performed to detect Ki-67 expression using a rabbit polyclonal anti-Ki-67 antibody (Novocastra, Newcastle, UK). Four representative sections were obtained from both *H. pylori*-infected and uninfected control cells for Ki-67 analysis. Sections were de-embedded and processed according to the instructions of the N-Histofine Simple Stain MAX-PO(R) kit (NICHIREI BIOSCIENCES Inc., Tokyo, Japan). Antigen retrieval was performed as follows. Before incubation with the blocking reagent, the sections were soaked in antigen retrieval reagent (DAKO) for 40 min at 95 °C and then cooled at room temperature for 15 min. The sections were then washed three times with PBS for 5 min. Data were analyzed and compared using the labelling index (LI), which is expressed as the percentage of nuclei with signals among 500 nuclei in a randomly chosen area.

Expression of phospho-Akt, phospho-c-Met, and β-catenin in 3D formation hepatocytes was detected using immunofluorescence staining on paraffin-embedded and formalin-fixed 3D cell culture Sects. (4 μm thick). Fluorescence immunohistochemistry was performed with rabbit monoclonal anti-phospho-Akt antibody (Cell Signaling Technology,Cat. no. 4060; Danvers, MA, USA), rabbit monoclonal anti-phospho-c-Met (Cell Signaling Technology, Cat. No. 3077, Danvers, MA, USA), and mouse monoclonal anti-β-catenin antibody (BD Transduction Laboratories, Cat. No. 610153, Franklin Lakes, NJ, USA). Cells incubated in the presence or absence of *H. pylori* were analyzed in four independent samples. Immunofluorescence was performed using the same methods for Ki-67 detection described above, except for the secondary antibody incubation step. The following secondary antibodies were used for IHC to detect β-catenin instead of secondary antibodies in the HISTOFINE kit: fluorescein-conjugated AffiniPure goat anti-mouse IgG or anti-rabbit IgG (Jackson ImmunoResearch Laboratories, Inc., West Grove, PA, USA). In all immunofluorescence assays, the nuclei were stained with DAPI to facilitate cell identification.

### Quantitative real-time PCR (qRT-PCR)

To evaluate the mRNA expression of inflammatory cytokines (e.g., *TNF* and *IL8)*, the signaling-related transcription factor *ETS1*, and the cell proliferation marker *PCNA,* Quantitative Real-Time PCR (qRT-PCR) was performed. Three independent samples from cells incubated with or without *H. pylori* were examined. Total RNA was extracted from 5–10 µm–thick specimens of frozen cell culture using RecoverAll Total Nucleic Acid Isolation Kit for FFPE (Ambion, Austin, TX, USA), following the provided instructions and stored at −80 ºC. cDNA was prepared from the total RNA using a high-capacity cDNA reverse transcription kit (Applied Biosystems, Foster City, CA, USA). qRT-PCR was performed with TaqMan Gene Expression Assays (Applied Biosystems, Waltham, MA, USA) with TaqMan Gene Expression Master Mix (Applied Biosystems, Waltham, MA, USA), following the instructions provided. The probe for *GAPDH* was used as an internal control. The PCR cycling program consisted of 40 cycles of 95ºC for 1 s and 60ºC for 20 s using the StepOne RT-PCR systems (Applied Biosystems, Waltham, MA, USA) following the holding step with 95ºC for 20 s. The data were analyzed using comparative ΔΔCt methods [18], comparing the *H. pylori*-infected and uninfected control groups. Comparative ΔΔCt methods calculates the expression fold change relative to a control group set as 1. TaqMan Gene Expression Assay ID for each target factor are listed below: *TNF;* Hs00174128_m1, *PCNA*; Hs00901425_m1, *IL8;* Hs00696862_m1, *ETS1*; Hs99999034_m1. *GAPDH*; Hs99999905_m1.

### NF-κB activity assay

NF-κB activation of 3D formation hepatocytes incubated with or without *H. pylori* was measured and quantified by Chemiluminescence Assay using Trans AM^™^ NF-κB p65 Chemi (Active Motif, Cat. No.40097, Carlsbad, CA, USA). Six frozen samples were obtained from each group and subjected to analysis. Nuclear extract was extracted from a 0.05 g frozen sample of 3D formation hepatocytes with cellulose beads using a Nuclear Extract Kit (version C4; Active Motif Cat. No.40010; Carlsbad, CA, USA) according to a standard protocol. The positive control cell extract (the Jurkat nuclear extract) provided by Trans AM^TM^NF-kB p65 Chemi was used to confirm the assay accuracy. Duplicate of 2 ug each nuclear extract sample were measured, and the average of the two values was calculated.

### Statistical analysis

Data are reported as mean ± standard error and were evaluated using Student’s *t*-test. Statistical analyses were performed using SigmaPlot version 10 (SYSTAT Software Inc., San Jose, CA, USA). Statistical significance was set at *P* < 0.05.

## Results

### *H. pylori* colonizes and persists in the 3D artificial liver model

H and E staining and IHC analysis with an anti-*H. pylori* antibody confirmed the successful establishment of the experimental setup and the infection of 3D cultured hepatocytes with *H. pylori*.

**Fig. 1:**
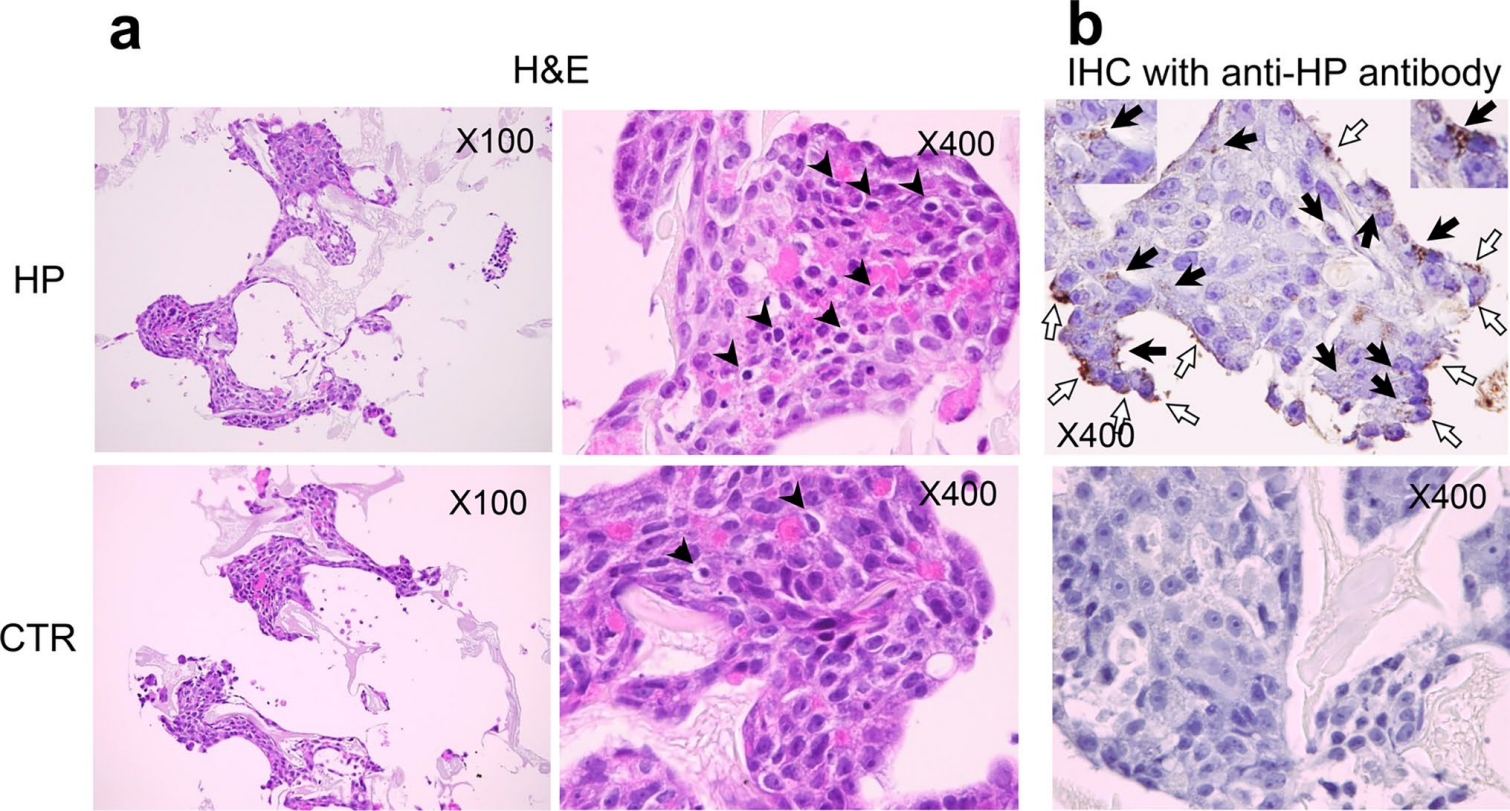
Construction of a 3D artificial liver model and subsequent infection with *H. pylori*. The top row shows cells incubated with *H. pylori* (HP), and the bottom row shows control (non-infected) cells (CTR). Representative images of three samples randomly selected from the inlet and outlet of the RFB reservoir are shown. Magnifications are indicated in the upper-right or lower-left corners (× 200 and × 400). **a **Hematoxylin and eosin staining showing hepatocytes attached to each other, forming a mass with cellulose beads. Cell shrinkage with pyknotic nuclei was observed (arrowheads) in both HP and CTR. **b** Immunohistochemistry with an anti-*H. pylori* antibody. *H. pylori* is identified by brown DAB staining. Following HP infection, numerous *H. pylori* are attached to hepatocytes on the surface of the cell mass (white arrows) and are localized within intracellular and/or intercellular spaces (black arrows). In CTR, no *H. pylori* are observed, confirming the absence of contamination. Magnified views of *H. pylori* invading intracellular and intercellular spaces are shown in the upper left and right corners for better visualization

The structural integrity of the 3D-reconstructed artificial liver was evaluated using hematoxylin and eosin (H and E) staining. Because no significant histological differences were observed between the inlet and outlet sites, representative images are presented in Fig. 1. Low-magnification images (**× **100) show the overall morphology of the 3D cultured hepatocytes (Fig. 1a), while high-magnification images (**× **400) reveal the detailed internal architecture of the 3D cultured hepatocytes (Fig. 1a). H and E staining demonstrated that the hepatocytes were attached to each other with cellulose beads, forming a hepatocyte mass in both cells with and without *H. pylori* incubation (Fig. 1a). Some pyknotic nuclear and cell shrinkage, characteristics of apoptosis, were observed both in cells incubated with/without *H. pylori*. Immunohistochemistry (IHC) was performed to evaluate the successful colonization of *H. pylori* within the 3D-reconstructed artificial liver. IHC with an anti-*H. pylori* antibody showed numerous *H. pylori*-attached hepatocytes on the surface of the hepatocyte mass (Fig. 1b). Notably, *H. pylori* was observed within the interior of the 3D-cultured hepatocyte aggregates. Given that these are 3D-cultured constructs, the presence of *H. pylori* within the cell mass in sections suggests that it is localized either in the intercellular spaces or intracellularly. These observations confirmed that *H. pylori* persisted in infection during 3D-constructed artificial liver culture, despite the circulation and exchange of the culture medium. *H. pylori* infection is rarely observed inside a mass. No *H. pylori* was detected in the control cell aggregates, ruling out potential contamination of the control sample.

### Ultrastructural evidence of *H. pylori* attachment, internalization, and intercellular infiltration

**Fig. 2:**
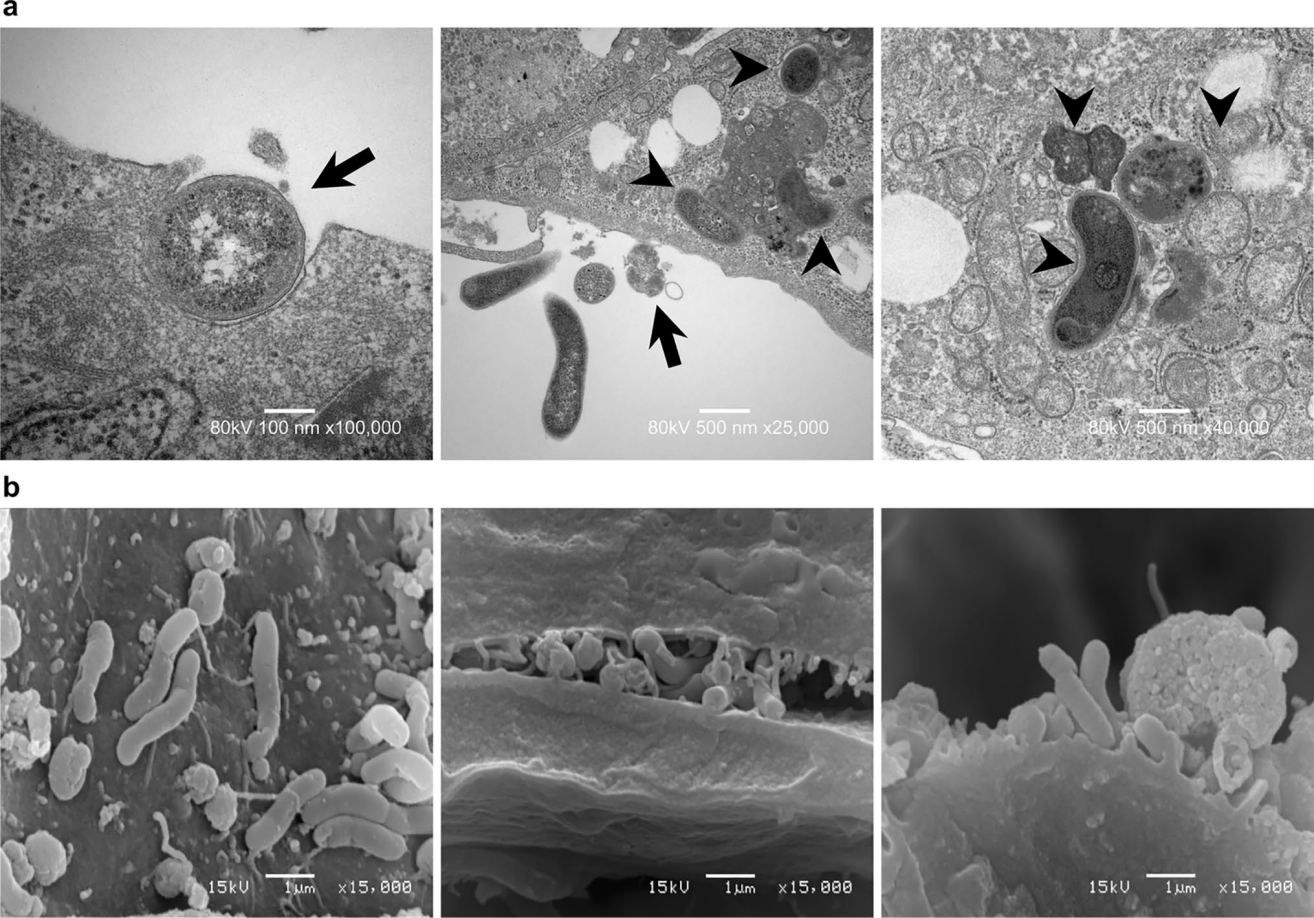
Adhesion, intercellular infiltration, and intracellular invasion of *H. pylori* into hepatocytes of an artificial liver observed by electron microscopy. **a** Transmission electron microscopy showing *H. pylori* attached (arrows) to the surface of hepatocytes with infiltration (arrowheads) of the intercellular space. Scale bars represent 100 nm in the left-most image and 500 nm in the middle and right-most images.** b** Scanning electron microscopy images demonstrating hepatocytes wrapped around *H. pylori*. *H. pylori* were found to invade and reside in the intercellular spaces and on the surface of the sterically constructed cell mass. Samples were collected from the inlet and outlet of the reservoir (*n* = 2 per site) for both the *H. pylori*-infected and non-infected cell groups. Scale bars represent 1 µm

Transmission electron microscopy (TEM) revealed *H. pylori* adhering to the hepatocyte surface and, in some instances, undergoing intracellular invasion (Fig. 2a). At the sites of attachment, cell surface remodeling was observed, characterized by the formation of cup-like structures of the plasma membrane that appeared to envelop the bacteria. Scanning electron microscopy (SEM) further demonstrated numerous *H. pylori* adhering to the cell surface, particularly within the intercellular clefts of the three-dimensional cell clusters (Fig. 2b). Notably, the bacteria in these locations predominantly maintained a bacillary morphology rather than transitioning to the coccoid form, which is typically associated with a dormant state under unfavorable environmental conditions. This suggests that the bacteria remained metabolically active within the microenvironment of the artificial liver model.

### *H. pylori* promotes hepatocyte apoptosis and modulates survival signaling pathways

**Fig. 3:**
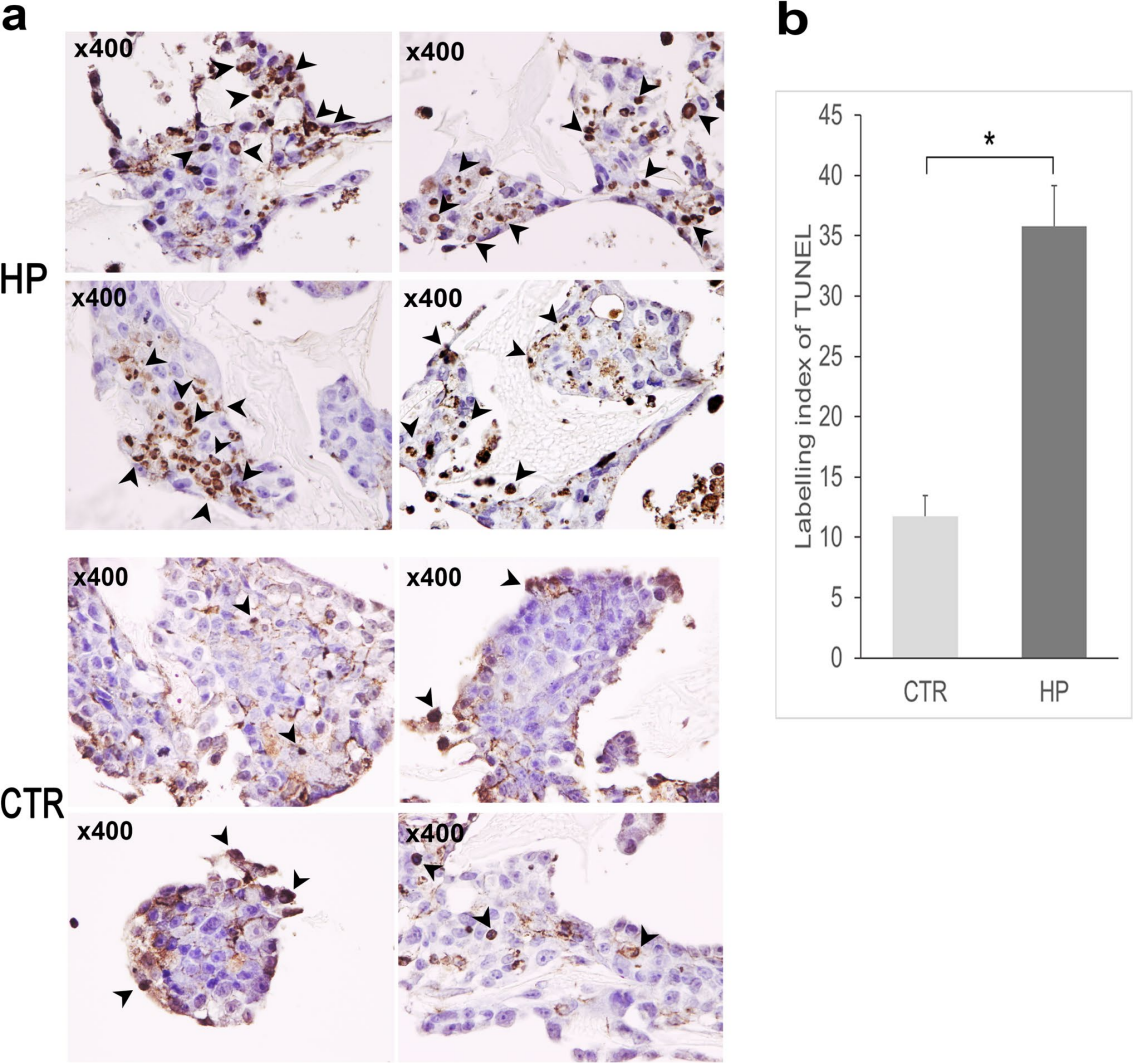
TUNEL assay of apoptosis in *H. pylori*-infected and uninfected 3D-constructed artificial livers. **a** TUNEL assay results for all examined samples. The top panels show four samples of 3D-constructed artificial livers incubated with *H. pylori* (HP), and the bottom panels show four control samples (CTR). Representative TUNEL-positive cells characterized by brown nuclear staining are indicated by arrowheads. TUNEL-positive cells (brown) are distributed non-uniformly throughout both the peripheral and interior regions. Magnification: × 400. **b** Quantification of the TUNEL assay results. The labeling index (LI) of TUNEL-positive cells was significantly higher in HP than in CTR. Data are presented as mean ± SD. *P < 0.05

To evaluate the impact of *H. pylori* infection on cellular homeostasis and protein expression within the 3D liver model, we first examined markers of cell turnover and intracellular signaling. TUNEL assay exhibited an increase in apoptotic cells within the 3D-constructed artificial livers following *H. pylori* infection (Fig. 3a). Quantitative analysis confirmed that the apoptotic index (LI) was significantly higher in *H. pylori*-infected 3D artificial livers (LI = 35.8 ± 3.4) than in uninfected controls (LI = 11.8 ± 1.7; *P* = 0.002; Fig. 3b).

**Fig. 4:**
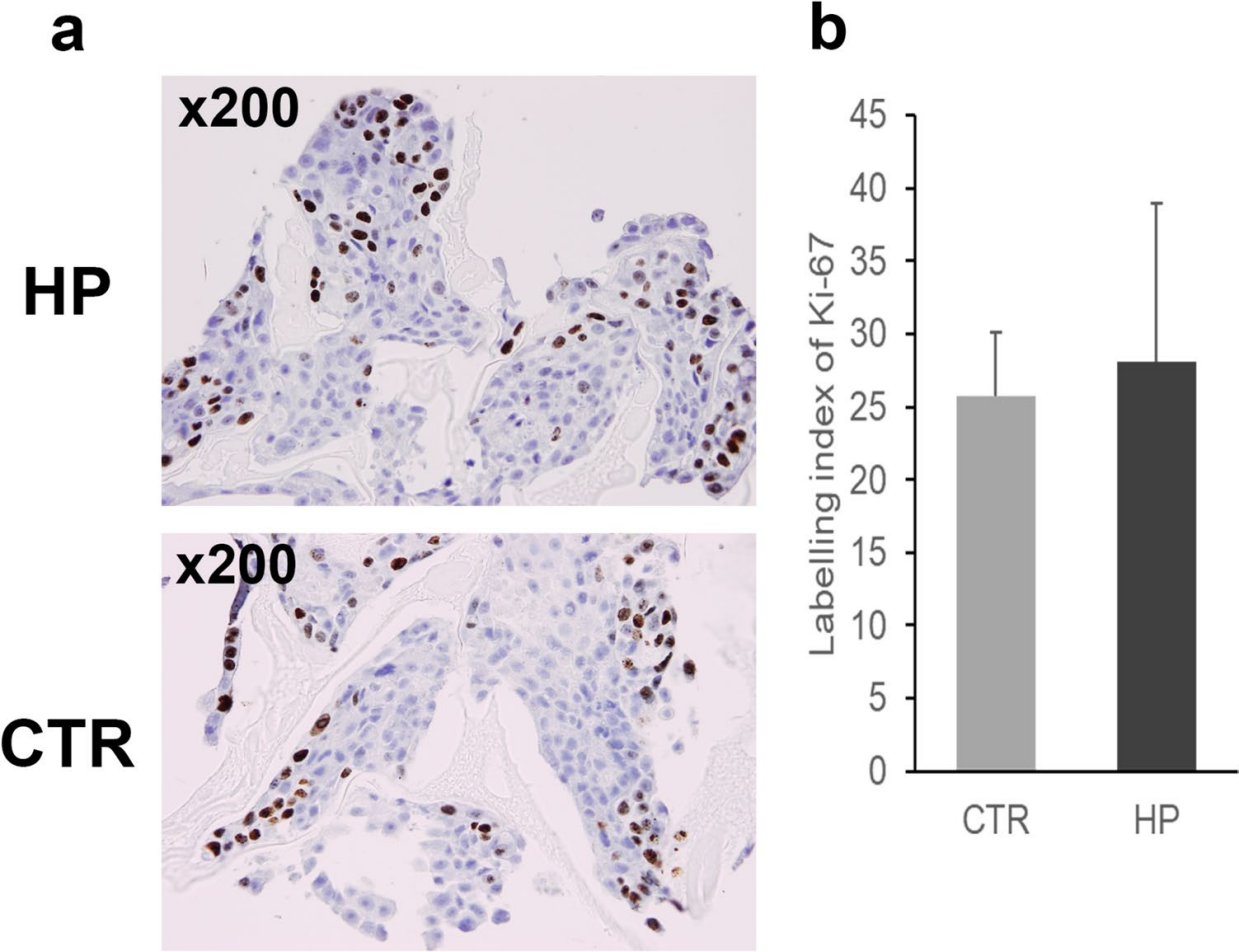
Ki-67 expression in *H. pylori*-infected 3D artificial livers. **a**  Representative images of Ki-67 immunostaining (*n* = 4). The top panel shows the *H. pylori*-infected condition (HP), and the bottom panel shows the uninfected control (CTR). Ki-67-positive nuclei (brown) appear as focal aggregates distributed non-uniformly throughout the 3D construct. Magnification: × 200. **b** Quantification of the Ki-67 positive cells. The y-axis represents the labelling index (LI). No significant difference was observed between HP and CTR (*P* = 0.426). Data are presented as mean ± SD

IHC analysis for Ki-67 revealed that the cells in the 3D-constructed artificial liver maintained their proliferative capacity, with Ki-67-positive nuclei observed regardless of *H. pylori* infection (Fig. 4a). The number of Ki-67-positive cells (Fig. 4b) was comparable between the *H. pylori*-infected group (LI = 28.1 ± 10.9) and the uninfected control group (LI = 25.7 ± 4.5) (*P* = 0.426), suggesting that *H. pylori* infection did not augment cell proliferation in the artificial liver.

**Fig. 5:**
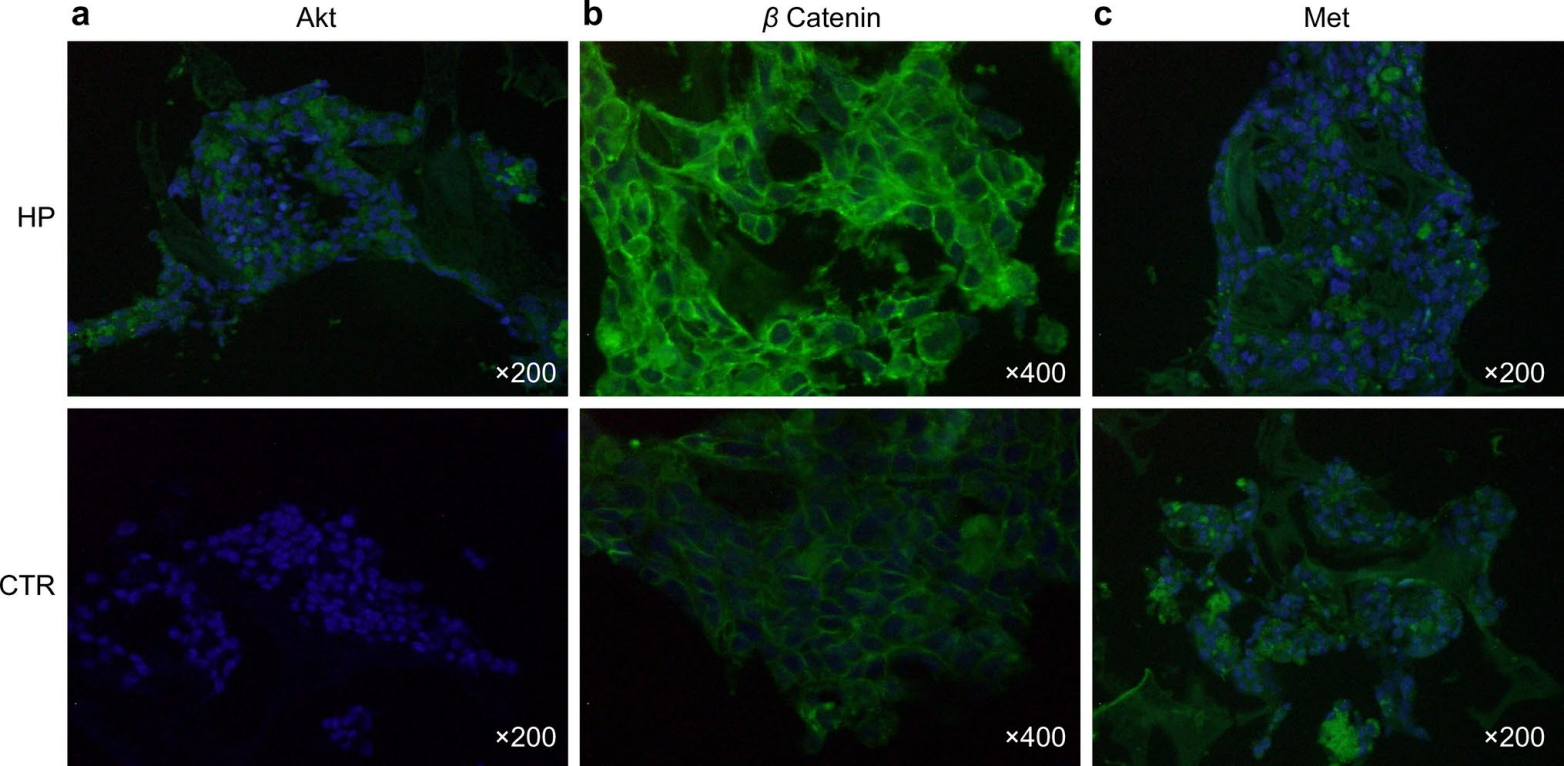
Expression of Akt, β-catenin, and c-Met following *H. pylori* infection. Immunofluorescence staining for **a** p-Akt (× 200), **b** β-catenin (× 400), and **c** p–c-Met (× 200) in 3D-cultured hepatocytes are shown. Artificial liver was incubated with (HP) or without (CTR) *H. pylori*. Proteins are stained green, and nuclei are counterstained blue with DAPI. Representative images of each condition are shown (*n* = 4). In the HP condition, p-Akt and β-catenin were observed to be localized in the cytoplasm, whereas p–c-Met expression showed no marked difference compared with CTR

Fluorescence-IHC analysis revealed the presence of phosphorylated Akt (p-Akt) in the cytoplasm of 3D hepatocytes following incubation with *H. pylori* (Fig. 5a). This finding indicates that *H. pylori* infection triggers Akt activation in this model. Fluorescence-IHC revealed that β-catenin was primarily localized in the cytoplasm of *H. pylori*-infected cells, with increased staining intensity compared to control cells. (Fig. 5b), Further, Fluorescence-IHC staining showed no significant difference in the intensity of phosphorylated c-Met between incubation of 3D formation hepatocytes with *H. pylori* and the control (Fig. 5c).

### Transcriptional alterations of inflammatory and growth-related genes

**Fig. 6:**
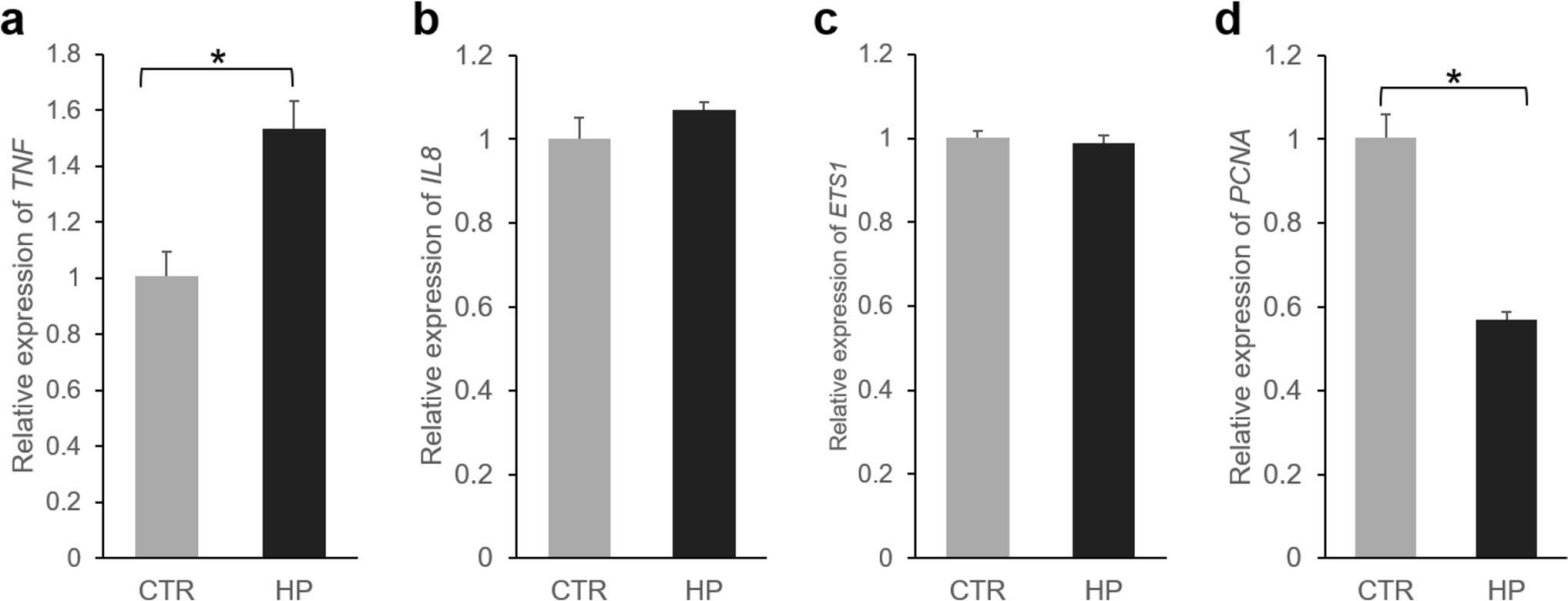
Quantitative analysis of inflammatory and proliferative gene expression. Relative mRNA expression levels of **a** *TNF*, **b** *IL8*, **c** *ETS1*, and **d** *PCNA* in cells incubated with (HP) or without (CTR) *H. pylori* were analyzed by quantitative RT-PCR. *GAPDH* was used as an internal reference. Samples were analyzed in triplicate for each condition. The y-axis represents the relative expression levels normalized to CTR. *TNF* expression was significantly increased, while *PCNA* expression was significantly decreased in HP compared with CTR. No significant differences were observed in *IL8* (*P* = 0.309) or *ETS1* (*P* = 0.633) expression between HP and CTR. Data are presented as mean ± SD. *P < 0.05

We next performed qRT-PCR analysis to quantify the mRNA expression levels of key genes involved in inflammation and cellular proliferation. To evaluate the multi-faceted impact of *H. pylori* on hepatocytes, we analyzed markers ranging from inflammatory cytokines (TNF-α and IL-8) to early oncogenic signaling (Ets-1) and DNA replication and repair (PCNA). qRT-PCR results (Fig. 6a) confirmed significantly higher levels (approximately 1.5-fold change) of *TNF* in *H. pylori*-infected cells (1.53 ± 0.10) than they did in non-infected controls (1.01 ± 0.09; *P* = 0.019), suggesting that *H. pylori* induced an inflammatory response characterized by *TNF* expression, which may contribute to the upregulation of apoptosis. Nevertheless, *IL8* levels were similar between *H. pylori* infection (1.07 ± 0.02) and control (1.00 ± 0.05) (*P* = 0.309) (Fig. 6b), suggesting that *H. pylori* did not induce the expression of key inflammatory cytokines in the artificial liver, in contrast to its effects in the stomach. *H. pylori* did not change *ETS1* levels (*H. pylori* infection; 0.99 ± 0.02 vs control; 1.00 ± 0.02; *P* = 0.633) (Fig. 6c), suggesting the absence of molecular markers associated with the very early stage of hepatocarcinogenesis. Notably, while Ki-67 protein expression remained unchanged as observed earlier, *PCNA* mRNA levels were significantly decreased in *H. pylori*-infected cultures (0.569 ± 0.02 vs. 1.00 ± 0.06; *P* = 0.002) (Fig. 6d). This suggests that *H. pylori* may impair DNA maintenance and repair capacity rather than simply suppressing the cell cycle.

### *H. pylori* colonization triggers NF-κB activation in 3D hepatocytes

**Fig. 7:**
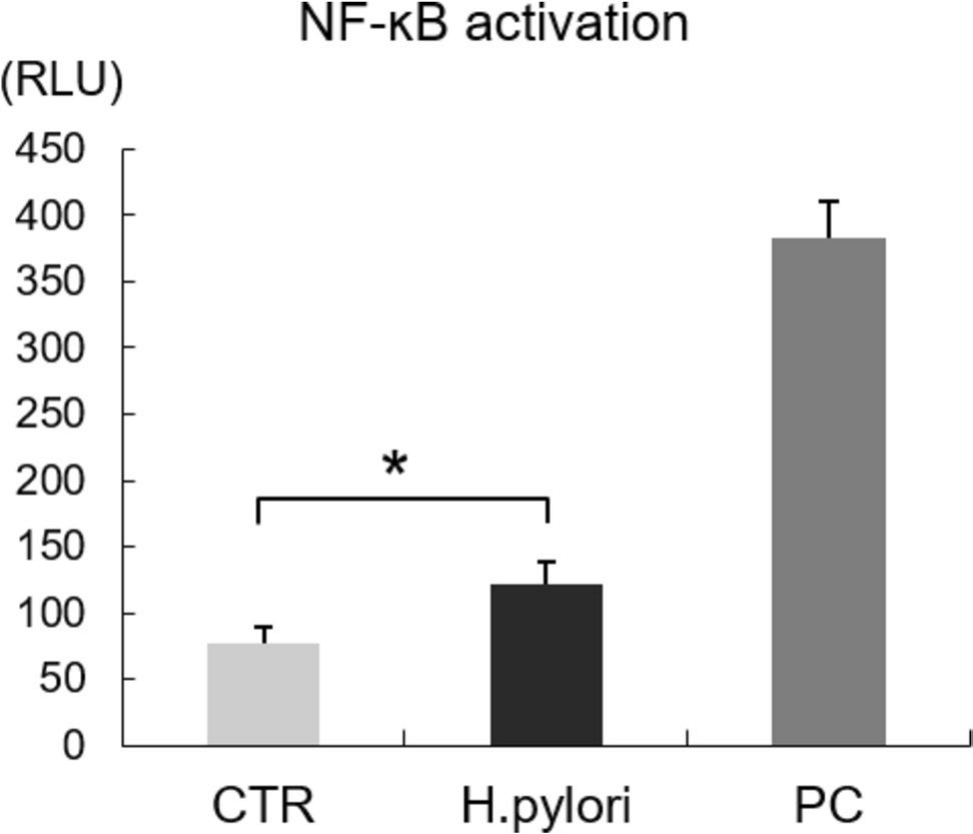
Comparison of NF-κB activation levels. NF-κB p65 activation in cells incubated with *H. pylori* (HP) and non-infected control (CTR) cells, measured by a chemiluminescent transcription factor assay, (*n* = 6 per condition). Jurkat nuclear extract was used as a positive control (PC) to validate the assay. The y-axis represents the chemiluminescence intensity in relative light units (RLU). NF-κB activation was significantly higher in HP than in CTR. Data are presented as mean ± SD *P < 0.05

Finally, we assessed the activation status of NF-κB, a master regulator of the inflammatory response, using a chemiluminescent DNA-binding assay. Significantly higher levels (approximately 1.6- fold change; *P* = 0.005) of NF-kB accumulation were detected in *H. pylori*-incubated cells (121.6 ± 17.3 RLU) than in controls (76.7 ± 12.6 RLU) (Fig. 7), confirming that *H. pylori* infection activates the NF-κB signaling pathway in 3D-cultured hepatocytes.

## Discussion

The incubation of *H. pylori* with 3D-formed hepatocytes clarifies how it infects human hepatocytes. Electron microscopy demonstrated that *H. pylori* attaches to the hepatocyte surface and enters hepatocytes. Our previous in vitro study demonstrated *H. pylori* invasion into hepatocytes [12], and the present findings support these results. Here, *H. pylori* occupied intercellular spaces, suggesting a loss of cell-to-cell connections that facilitates entry via this intercellular route. Since the bacteria remained in the bacillus form rather than converting to the coccoid form—the defensive morphology adopted to survive when environmental conditions are unfavorable—*H. pylori* might not only attach to the surface of the liver to invade the intracellular space, but may also loosen intercellular junctions and invade intercellular spaces, thereby escaping from unfavorable extracellular conditions and facilitating survival. *H. pylori* usually inhabits human stomachs containing gastric juice. One of the reasons *H. pylori* can survive in an acidic environment is its production of urease, which neutralizes acids. Another reason may be its ability to move through the gaps between cells and internalize into hepatocytes to avoid a difficult extracellular environment. In the liver, which is an organ under rigorous immunological surveillance, the ability to invade the intercellular space represents a critical survival strategy for *H. pylori*. Occupation of this niche would enable *H. pylori* to evade Kupffer cells and other innate immune mechanisms while limiting exposure to complement proteins and additional antimicrobial factors present in the circulation. The extracellular matrix and adjacent cellular surfaces would support *H. pylori* adhesion and localized proliferation. Furthermore, intimate contact with hepatocytes would allow bacteria to modulate host signaling pathways, thereby attenuating inflammatory responses and promoting conditions favorable to continued infection.

The redistribution of β-catenin observed in our model likely reflects the molecular basis for the loosening of intercellular junctions described above. In hepatocytes infected with CagA-positive *H. pylori*, β-catenin was found to be localized within the cytoplasm, whereas its expression was negligible in the control group. This cytoplasmic accumulation is a recognized consequence of CagA-mediated disruption of intercellular complexes [19]. This finding is consistent with our electron microscopy observations showing *H. pylori* occupying loosened intercellular spaces, suggesting that the shift of β-catenin to the cytoplasm serves as a molecular signature of junctional disturbance that facilitates bacterial niche occupation. These changes appear to occur independently of the c-Met pathway, as evidenced by our data showing no significant change in c-Met expression.

Our findings highlighted an apparent paradox between the activation of pro-survival pathways and the induction of apoptosis. *H. pylori* infection triggered a robust inflammatory response, evidenced by the significant upregulation of TNF-α and the activation of NF-κB. While both NF-κB and Akt typically function as pro-survival and anti-apoptotic signals, their activation in this model was evidently insufficient to override the potent pro-apoptotic stimuli induced by *H. pylori*-related stress and *TNF-α* signaling. Based on these results, the activation of these pathways in our 3D-artificial liver model may represent an attempted but failed compensatory survival response that does not reach the threshold necessary to protect hepatocytes from undergoing apoptosis, as indicated by the marked increase in TUNEL-positive cells.

Similarly, the discrepancy between the unchanged Ki-67 labeling index and the significant decrease in *PCNA* mRNA expression characterizes the specific stress state of the infected hepatocytes. While Ki-67 is a strict marker of active cell proliferation, PCNA plays a dual role, being essential for both DNA replication and DNA repair mechanisms [20]. Our data show that while *H. pylori* infection does not trigger an active proliferative response (as indicated by stable Ki-67 levels), it significantly suppresses *PCNA* expression. This downregulation suggests that *H. pylori* impairs the genomic maintenance and DNA repair capacity of hepatocytes. Therefore, the reduction in PCNA*,* combined with the increase in apoptosis, indicates that the infected hepatocytes are suffering from a compromised ability to repair cellular damage, leading to a state of uncompensated injury rather than active regeneration.

Moreover, our model revealed distinct differences in the inflammatory signaling architecture compared to that typically observed in the gastric mucosa. In gastric epithelial cells, *H. pylori* infection is known to directly trigger the secretion of high levels of IL-8 via NF-$\kappa$ B activation. However, in our 3D-hepatocyte model, *IL8* mRNA levels remained unchanged despite the significant activation of NF-$\kappa$ B and the upregulation of *TNF* mRNA levels. This “uncoupling” of NF-$\kappa$ B activation from IL-8 induction suggests that the typical chemokine-driven inflammatory circuit may not be the primary driver of *H. pylori*-associated hepatocyte injury. Similarly, the absence of changes in *ETS1* and c-Met expression indicates that the observed cellular stress occurs independently of these conventional pathogenic pathways. Thus, *H. pylori* appears to modulate hepatocyte biology through a unique pathological axis, where the response is skewed toward a TNF-$\alpha$/NF-$\kappa$ B-mediated pro-apoptotic state.

Our findings clarify that *H. pylori* is capable of infecting human hepatocytes within a 3D-culture environment and triggering significant pathological responses, including TNF-α/NF-κB-mediated inflammation, apoptosis, and the redistribution of β-catenin. *H. pylori* is an ancient pathogen that has evolved sophisticated strategies to evade host immune systems and maintain persistent infection [21–23]. While a direct causal link to hepatocellular carcinoma remains to be fully established, the ability of *H. pylori* to loosen intercellular junctions and occupy the intercellular niche suggests a potential mechanism for chronic liver injury if the bacteria translocate via biliary or portal routes. This study provides one possible answer to whether *H. pylori* can directly affect hepatocyte biology, highlighting its role in creating a pro-inflammatory and pro-apoptotic environment—conditions that are recognized precursors to longer-term pathological changes. Nonetheless, while our 3D artificial liver model provides valuable insights, further studies are warranted to clarify whether these effects occur in the human liver in vivo.

The main limitation of this study is the use of an artificial liver model, potentially limiting real-life applicability. Nevertheless, compared with other in vitro methods for studying pathological conditions, RFB is advantageous because it facilitates cell culture 3D construction. Additional studies involving clinical populations are warranted to confirm the clinical validity of our findings. Another limitation is the use of a single cancer cell line (FLC-4) and a single *H. pylori* strain (NCTC11637), which may not be representative of all HCCs or infections. Further clarification of our research findings requires studies using the *H. pylori* strain alongside various other cell lines.

## Data Availability

The datasets generated and/or analyzed during the current study are available from the corresponding author on reasonable request.
